# GIST suture-line recurrence at a gastrojejunal anastomosis 8 years after gastrectomy: can GIST ever be described as truly benign? A case report

**DOI:** 10.1186/1477-7819-8-90

**Published:** 2010-10-14

**Authors:** Alexandros Papalambros, Athanasios Petrou, Nicholas Brennan, Kostantinos Bramis, Evangelos Felekouras, Efstathios Papalambros

**Affiliations:** 1 Department of Pathology, University of Athens, Medical School, Greece; 2 Department of Hepatobilary Surgery, Churchill Hospital, Oxford, UK; 3 First Department of Surgery, University of Athens Medical School, Greece; 4 Professor of Surgery, University of Athens Medical School, Greece

## Abstract

We present the case of a 71 year old man with recurrence of a Gastro Intestinal Stromal Tumour (GIST) at the gastrojejunal anastomosis eight years following partial gastrectomy for a very small primary gastric GIST. He presented acutely on both occasions with haemodynamic shock secondary to massive haematemesis. During his initial presentation in 2001, an emergency laparotomy was performed, demonstrating a pre-pyloric ulcerative lesion. The histopathology was in keeping with a diagnosis of a gastric GIST with a < 2 cm tumour, with <5 mitosis per 50/HPF, no signs of necrosis and invasion limited to the mucosa. Eight years later the same patient presented with a similar clinical picture of haemodynamic instability secondary to haematemesis. Emergency endoscopy showed an irregularly shaped elevated lesion on the gastrojejunostomy line suggestive of recurrence. He subsequently underwent completion gastrectomy and the histology revealed a 0.8 cm GIST tumour composed of spindle cells with <5 mitosis per 50/HPF, tumor invasion into the submucosa and positive expression of c-kit and SMA. The patient remains recurrence free 18 months post surgery. The literature suggests that tumour size, mitotic rate and tumour site are the most important predictive factors of recurrence. Additional features such as the presence of necrosis, local tumour invasion and positive resection margins, can also influence recurrence rates. In this case the lesion was a gastric GIST, very small (<2 cm), had low proliferation rate (<5 mitosis/HPF), lacked necrosis and was limited to the mucosa. Recurrence of such a primary GIST at the anastomotic line, eight years after initial resection has never been demonstrated among review of several thousand primary GISTs. This case highlights how even the most innocent GISTs can never be described as truly benign.

## Background

Gastrointestinal stromal tumours (GISTs) are the most common form of mesenchymal tumours found in the gastrointestinal (GI) tract. GISTs most commonly occur in the stomach and small intestine but can also be found in smaller numbers in the colon, rectum and oesophagus [1]. Many GISTs are asymptomatic and are discovered incidentally, however over half of gastric GISTs present with signs of GI bleeding and anaemia with a smaller proportion presenting with abdominal pain or as an abdominal mass [2]. Histologically, GISTs are often composed of spindle shaped cells with a smaller number dominated by epithelioid or a mixture of both spindle and epithelioid cells [3,4]. Although GISTs are a relatively newly discovered cancer, there has been increased attention due to the development of effective targeted agents [5]. Tyrosine kinase inhibitor (TKI) therapy with imatinib has significantly prolonged progression free survival in advanced unresectable disease with over 80% of advanced GIST patients benefiting [5].

Primary GISTs have uncertain malignant potential and the long term prognosis of GIST has been challenging for clinicians and pathologist alike. Large multi centre studies on primary GISTs have lead to the development of prognostic scoring systems based on tumour histopathology [6,7]. Within these studies, several thousand primary GIST cases have been reviewed and none of them have demonstrated late local recurrence of a very small (<2 cm), low mitotic rate (<5 mitosis/50 High Power Field (HPF)) gastric tumour [3,4,6-9]. For this reason these tumours have been described as essentially benign [7,10]. In this report we discuss recurrence in such a case.

### Case Presentation

A 71 year old man with signs of syncope and haemorrhagic shock secondary to massive haemetemesis was referred for emergency treatment and investigation to the 1^st ^Department of Surgery, University of Athens Medical School in 2009. Eight years earlier the same patient, who had a known history of gastric ulcers, presented with a similar clinical picture to a different surgical unit. On admission he showed signs of haemorrhagic shock with a haemoglobin level (Hg) of 7 g dL. Emergency upper GI endoscopy was unable to identify the source of bleeding due to large volumes of blood in the stomach. Surgical treatment with a laparotomy was decided and the intraoperative findings demonstrated an acute gastric hemorrhage secondary to a massive propyloric ulcerative lesion. Resection of the lesion was decided and a distal gastrectomy and Billroth II reconstruction performed. The subsequent histology revealed a <2 cm gastrointestinal stromal tumour, with a mitotic rate of < 5 mitosis/50 per HPF, lacking necrosis and localized to the gastric mucosa. The patient made an uneventful recovery and was discharged eleven days post surgery. The patient was reviewed over the following two years and repeat endoscopies failed to reveal any signs of recurrence. The patient subsequently declined further surveillance and follow up.

At his readmission in 2009 the patient was primarily treated conservatively due to his hemodynamic instability. After successful resuscitation, an emergency upper GI endoscopy was performed which revealed an irregularly shaped elevated lesion on the gastrojejunostomy line and a thrombus at the center of the lesion. The hemorrhagic lesion was situated along the posterior anastomotic suture line. Multiple biopsies were performed and a definitive endoscopic haemostasis was obtained.

**Figure 1:**
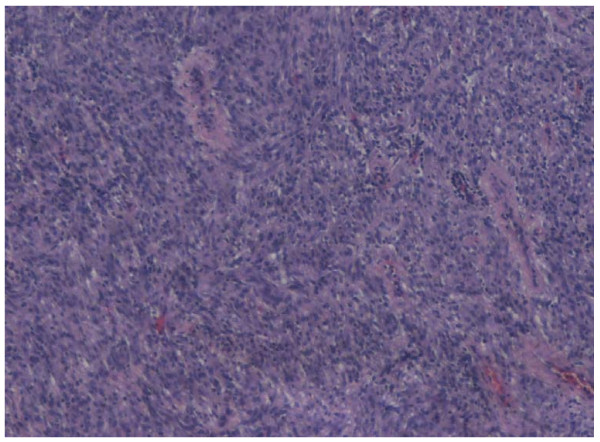
Gastric GIST in H-E stain (×20)

**Figure 2:**
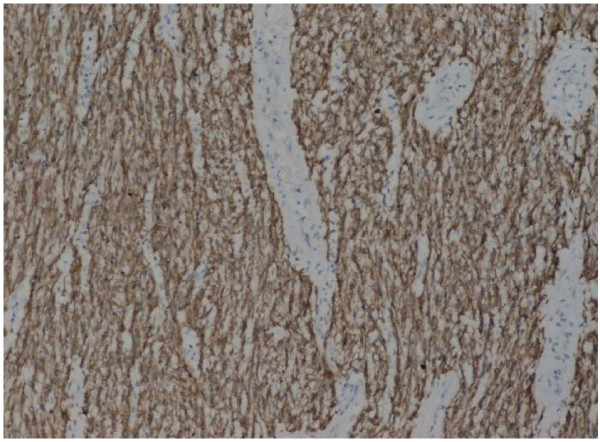
Gatric GIST/C-kit immunoexpression (×40)

Preoperative staging computed tomography (CT) showed no lymphadenopathy or hepatic metastasis and as the patient's performance status was otherwise excellent, the decision for a second operation was deemed favorable. The patient went on to have a successful completion gastrectomy with regional lymphadenectomy and the continuity of the gastrointestinal tract was maintained through the Roux-en-Y method. It is important to note that lymphadenectomy is not routinely performed in GIST as metastatic spread rarely occurs through the lymphatic system. However the unusual presentation of the case created uncertainty over the malignant potential of the tumour and the experienced surgeons deemed lymphadenectomy the most appropriate measure in this instance. Histological review of the specimen showed macroscopically an ulcerative lesion on the suture-line, measuring 0.8 cm in diameter. The cut surface was gray with a rubbery consistency. Microscopically, it was a gastrointestinal stromal tumor (figure 1), composed of spindle cells with mild to moderate nuclear pleomorphism. The stroma focally had a myxoid appearance. The tumor invaded into the submucosa, showed no signs of necrosis and had positive expression of c-kit (figure 2), focally positive expression of SMA, and negative expression of CD34. The postoperative course was uneventful, and the patient shows no evidence of recurrence 1 year and 6 months after the last surgery. It is noteworthy to mention that GIST in this patient occurred sporadically and that there were no clinical findings suggestive of familial GIST which can be seen in patients with neurofibramatosis type 1 (NF1) or in the Carney-Stratakis dyad.

## Conclusions

The significant majority of mesenchymal tumors of the stomach are believed to derive from the interstitial cells of Cajal, the gut pacemaker cells [11]. Since this cell expresses CD117, it was assumed that expression of CD117 by GIST was evidence of origin from that cell type [11]. GIST can occur anywhere in the gastrointestinal (GI) tract but most commonly occurs in the stomach. The median age of presentation is 60 years with no significant differences between males and females [12]. The presentation varies according to tumour site with GI bleeding and abdominal pain being most common [12]. Endoscopy with biopsy is used to identify the tumour with the definitive diagnosis depending on histological and immunohistochemical analysis. GISTs show a wide range of histologic appearances but are broadly divided into spindle and epithelioid cell types. In general, the risk of malignancy is greater in epithelioid tumors than in spindle-celled neoplasms [11,12]. The most important immunohistochemical markers of GISTs are expression of KIT (CD117), which is found in over 90 percent of GISTs, and CD34 which occurs in over 80 percent [11]. SMA is demonstrable in about 25 percent and a smaller number of GISTs (3% to 5%) have mutations in platelet derived growth factor receptor alpha (PDGFRA) instead [11]. Imatinib, a tyrosine kinase (TKI) inhibitor, antagonizes the effects of the KIT and PDGFRA proteins and has revolutionized the treatment of advanced and unresectable GISTs [5]. There is growing evidence that responsiveness to TKI inhibitors is dependent on the type and site of mutation with deletions appearing to be more aggressive than point mutations and exon 9 mutations showing less responsive to imanitib therapy than exon 11 lesions [5].

Primary GISTs have the potential for curative treatment, with surgical resection the first line option for all resectable non metastatic tumours. The overall 5 year survival rate for resectable GISTs has been shown to range from 46% to 78.5% [3,4]. However, predicting the recurrence rate of primary resectable GISTs has been very challenging. Over the past decade there have been several high profile risk stratification tools for predicting recurrence rates. The National Institute for Health (NIH) and the National Comprehensive Cancer Network (NCCN) has developed risk schemes for primary GIST tumours [6,7,10]. The American Joint Committee on Cancer (AJCC) has created a similar scheme but also incorporate advanced and metastatic GISTs [13]. The latest risk scheme has recently been published in the seventh edition of the international union against cancer (UICC) where a novel classification and staging system using TNM is proposed [14].

The NIH risk scheme originally developed in 2002 by a consensus conference of experts was based on the tumour size and mitotic rate - subdividing GIST into very low risk (tumour < 2 cm, < 5 mitosis/50HPF), low risk (tumour 2-5 cm, < 5 mitosis/50HPF), intermediate risk (tumour 5 cm-10 cm, < 5 mitosis/50HPF or tumour < 5 cm and 6-10 mitosis/50HPF) and high risk (tumour > 5 cm, >5 mitosis/50HPF or tumour >10 cm and any mitotic rate) [6]. This prediction scheme was later validated with large population studies on GISTs. Nillson *et al *reviewed 288 patients with primary GIST and reported no recurrence in the very low risk group and a 1.9% recurrence in the low risk group [8]. Tryggvason *et al *performed a similar study and also demonstrated no recurrence in the very low risk group [9]. This risk stratification was further expanded by Miettinen and Lasota by including tumour site and this system was adopted by the NCCN [7,10]. Gastric GISTs had the lowest rate of recurrence with the highest rates in duodenal and rectal GISTs. In the largest ever series of GIST patients (actual data for over 1900 GIST patients) Miettinen and Lasota incorporated mitotic rate, tumour size and tumour location as predictors for tumour recurrence [7]. In the lowest risk group, tumour size <2 cm and < 5 mitosis/50HPF, there was no reported recurrence of GIST from any gastrointestinal site and this group was essentially considered benign. Tumour size <5 cm and < 5 mitosis/50 HPF (NIH very low risk score) carries a 1.9% risk of recurrence from gastric GIST increasing to 8.3% and 8.5% for duodenal and rectal GIST respectively. The TNM system proposed by the UICC applies a similar system to Miettinen and Lasota and categorizes tumors into four major T-categories and corresponding UICC stages. The main purpose of the TNM system is to produce a more standardized surgical and oncological treatment for patients with GIST. The usefulness of this system will become evident with future clinical studies.

There have been subsequent studies and case reports documenting late GIST recurrence with metastasis from small (>2 cm but < 5 cm) tumours but no reported cases, from our literature review, of local recurrence of a very small (<2 cm), < 5 mitosis/50HPF, gastric GIST [15]. Additional risk factors associated with recurrence include presence of necrosis, infiltration of neighbouring structures, high cellularity, serosal invasion, high vascularity and positive tumour margins [12]. The original primary GIST in this report was located in the stomach, very small (< 2 cm), < 5 mitosis/50HPF, showed no signs of necrosis, was localised to the mucosa and had negative tumour margins.

There are several plausible hypotheses for tumour recurrence in this instance. Despite the fact that the histopathological specimen resected was R0 there may still have been some local infiltration of the tumour margin. In addition there are several studies which highlight the risk of tumour recurrence with intraoperative tumour rupture or laceration [16]. Although this was not reported at the time of surgery it would be a reasonable explanation for recurrence of such a low risk GIST. Multiple sporadic GISTs have been described in patients who do not have germline mutations in KIT/PDGFRA or neurofibromatosis [17]. In this instance there would be development of an independent, potentially different histopathogically, GIST [17]. Unfortunately the original specimen is no longer available for further comparative analysis and this theory could not be further investigated.

According to the literature recurrence of GIST is dependent on tumour size, mitotic rate and tumour site, with additional factors such as necrosis, local invasion and tumour free margins influencing recurrence also. In the current case, the mass was very small, located in the stomach, exhibited very low mitotic activity, showed no signs of necrosis and was limited to the mucosa. Recurrence of such a GIST tumour on the suture line eight years after resection presents a previously undocumented case and demonstrates that even the most subtle GISTs can never be considered as truly benign.

## Consent

Written informed consent was obtained from the patient for publication of this case report and any accompanying images. A copy of the written consent is available for review by the Editor-in-Chief of this journal.

## Competing interests

The authors declare that they have no competing interests.

## Authors' contributions

APe and NB wrote the manuscript. KB, EF and EP where the surgical team and reviewed the manuscript. APa reviewed the pathology. All authors read and approved the final manuscript.
